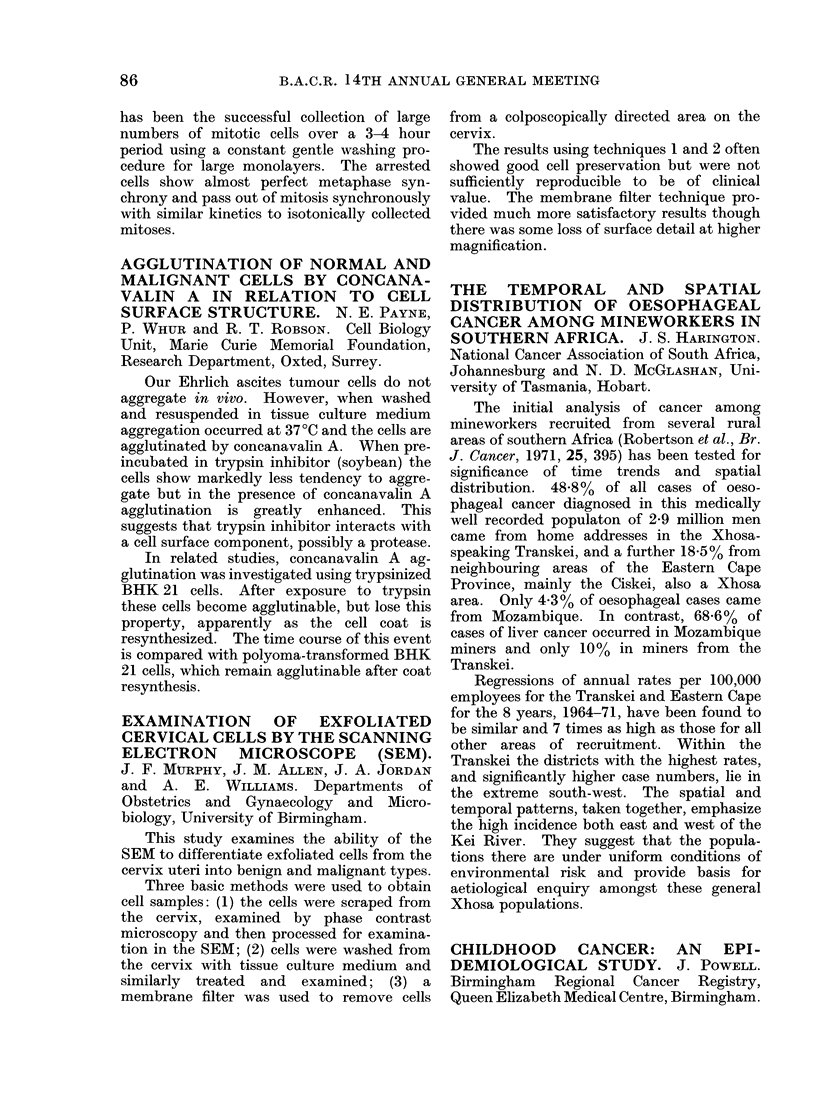# Examination of exfoliated cervical cells by the scanning electron microscope (SEM).

**DOI:** 10.1038/bjc.1973.108

**Published:** 1973-07

**Authors:** J. M. Allen, J. A. Jordan, A. E. Williams


### EXAMINATION OF EXFOLIATED CERVICAL CELLS BY THE SCANNING ELECTRON MICROSCOPE (SEM).

J. F. MURPHY, J. M. ALLEN, J. A. JORDAN and A. E. WILLIAMS. Departments of Obstetrics and Gynaecology and Microbiology, University of Birmingham.

This study examines the ability of the SEM to differentiate exfoliated cells from the cervix uteri into benign and malignant types.

Three basic methods were used to obtain cell samples: (1) the cells were scraped from the cervix, examined by phase contrast microscopy and then processed for examination in the SEM; (2) cells were washed from the cervix with tissue culture medium and similarly treated and examined; (3) a membrane filter was used to remove cells from a colposcopically directed area on the cervix.

The results using techniques 1 and 2 often showed good cell preservation but were not sufficiently reproducible to be of clinical value. The membrane filter technique provided much more satisfactory results though there was some loss of surface detail at higher magnification.